# Retraction Note: GPX8 is transcriptionally regulated by FOXC1 and promotes the growth of gastric cancer cells through activating the Wnt signaling pathway

**DOI:** 10.1186/s12935-024-03269-6

**Published:** 2024-02-22

**Authors:** Hong Chen, Lu Xu, Zhi-li Shan, Shu Chen, Hao Hu

**Affiliations:** 1 Department of Gastrointestinal Surgery, The First Affiliated Hospital of Soochow University, No.899, Pinghai Road, Suzhou, 215000 Jiangsu China; 2 Department of General Surgery, Suzhou Dushu Lake Hospital Affiliated to Soochow University, Suzhou, 215000 Jiangsu China; 3 Affiliated Hospital of Jiangsu University, Zhenjiang, Zhenjiang, 212000 China

**Cancer Cell International (2020) 20:596**

10.1186/s12935-020-01692-z

The Editors-in-Chief have retracted this article. Concerns were raised regarding a number of figures, specifically:

Figure 5h appears to overlap with Figure 4f in an article by different authors that was simultaneously under consideration with another journal [1].

Multiple figures, including Figures 2e, 3f and 5h appear to overlap with figures in a previously published article by different authors [2].

The Editors-in-Chief therefore no longer have confidence in the results and conclusions of this article. Hong Chen, Lu Xu, Shu Chen and Hao Hu disagree with this retraction. Zhi-li Shan did not respond to correspondence from the publisher about this retraction.
